# A Putative Gene *sbe3-rs* for Resistant Starch Mutated from *SBE3* for Starch Branching Enzyme in Rice (*Oryza sativa* L.)

**DOI:** 10.1371/journal.pone.0043026

**Published:** 2012-08-24

**Authors:** Ruifang Yang, Chunlong Sun, Jianjiang Bai, Zhixiang Luo, Biao Shi, Jianming Zhang, Wengui Yan, Zhongze Piao

**Affiliations:** 1 Crop Breeding and Cultivation Research Institute, Shanghai Academy of Agricultural Sciences, Shanghai, China; 2 Rice Research Institute, Anhui Academy of Agricultural Sciences, Anhui, China; 3 United States Department of Agriculture-Agriculture Research Services, Dale Bumpers National Rice Research Center, Stuttgart, Arkansas, United States of America; 4 College of Plant Science, Jilin University, Changchu, China; Lawrence Berkeley National Laboratory, United States of America

## Abstract

Foods high in resistant starch (RS) are beneficial to prevent various diseases including diabetes, colon cancers, diarrhea and chronic renal or hepatic diseases. Elevated RS in rice is important for public health since rice is a staple food for half of the world population. A *japonica* mutant ‘Jiangtangdao 1’ (RS = 11.67%) was crossed with an *indica* cultivar ‘Miyang 23’ (RS = 0.41%). The mutant *sbe3-rs* that explained 60.4% of RS variation was mapped between RM6611 and RM13366 on chromosome 2 (LOD = 36) using 178 F_2_ plants genotyped with 106 genome-wide polymorphic SSR markers. Using 656 plants from four F_3∶4_ families, *sbe3-rs* was fine mapped to a 573.3 Kb region between InDel 2 and InDel 6 using one STS, five SSRs and seven InDel markers. *SBE3* which codes for starch branching enzyme was identified as a candidate gene within the putative region. Nine pairs of primers covering 22 exons were designed to sequence genomic DNA of the wild type for *SBE3* and the mutant for *sbe3-rs* comparatively. Sequence analysis identified a missense mutation site where Leu-599 of the wild was changed to Pro-599 of the mutant in the *SBE3* coding region. Because the point mutation resulted in the loss of a restriction enzyme site, *sbe3-rs* was not digested by a CAPS marker for *Spe*I site while SBE3 was. Co-segregation of the digestion pattern with RS content among 178 F_2_ plants further supported *sbe3-rs* responsible for RS in rice. As a result, the CAPS marker could be used in marker-assisted breeding to develop rice cultivars with elevated RS which is otherwise difficult to accurately assess in crops. Transgenic technology should be employed for a definitive conclusion of the *sbe3-rs*.

## Introduction

Starch is the major dietary source of carbohydrates which is composed of two types of molecules, amylose (Am) and amylopectin (Ap) [1], [2]. Am is essentially a linear molecule composed of α (1,4)-linked glucosidic chains, whereas Ap is a highly branched glucan with α (1,6) glucosidic bonds for a connection of linear chains [3]. Based on the characteristics of enzymatic digestion, starch can be classified into rapidly digestible starch (RDS), slowly digestible starch (SDS) and resistant starch (RS) [4], [5]. RS is a small fraction of starch resistant to hydrolysis by exhaustive α-amylase and pullulanase treatment *in vitro* [5], and is defined as “the sum of starch and products of starch degradation not absorbed in the small intestine of healthy individuals” [6], [7].

RS has similar physiological functions to those of dietary fiber, and is completely resistant to enzymatic digestion in the human small intestine. As a result, RS reaches the large intestine where it acts as substrate for fermentation by microflora [8]. Short-chain fatty acids (SCFA) are major end products of the fermentation, and these acids are able to promote optimal function of the viscera [9]–[11]. Increasing RS consumption is becoming an effective means to improve nutrition for public health. Foods high in RS have the potential to prevent pathogen infections and diarrhea with benefits in various aspects, such as inflammatory bowel disease [12], colon cancer risk [13], insulin resistance and diabetes [14], and chronic renal or hepatic disease [15]. Diet-related noninfectious chronic diseases including coronary heart disease, certain cancers (especially of the colon and rectum), and diabetes are major causes of morbidity and mortality world wide [10]. These unique physical functions of RS have received increasing attention from plant researchers in recent years.

As the primary dietary source of carbohydrates in the world, rice (*Oryza sativa* L.) plays an important role in meeting energy requirements and nutrient intake in cereal crops. However, RS content is generally under 3% in hot cooked rice cultivars, which is not enough to confer the associated health benefits [16], [17]. Therefore, many studies have focused on an elevation of RS content in rice cultivars using mutation breeding and bioengineering. Many mutants with elevated RS content have been identified in rice, including Goami 2 [18], RS111 [19], and ‘Jiangtangdao 1’ [20]. ‘Teqing Resistant Starch’ (TRS) is another high amylose and RS transgenic line developed by modifying antisense RNA inhibition for starch branching enzymes (SBE) in rice [21]. Similarly, high RS mutants have been reported in wheat (*Triticum durum* L.) [22], maize (*Zea mays* L.) [11] and barley (*Hordeum vulgare* L.) [23].

Determination of starch digestibility is difficult, which has greatly hindered breeding progress for improving RS in crops. Ideally, the amount of RS is determined *in vivo* using techniques such as the human ileostomy model or intubation [24]. However, these approaches are problematic for various reasons, notably because they are laborious or invasive and expensive. Sometimes, the humans involved in the experiments may be at risk. In fact, such *in vivo* techniques are neither feasible nor practical in many laboratories. Therefore, most analysis of RS is conducted *in vitro* to spectrophotometrically determine the RS after enzymatic reaction. However, the physiological relevance of *in vitro* determined RS to *in vivo* determined RS is questionable [24], [25]. There are some evidences to show that *in vitro* assays do not quantitatively measure RS as the *in vivo* assays do [26], [27].

Analytical difficulties for determining RS could be overcome with the use of marker-assisted selection in breeding programs. Breeding materials in the early generations can be screened with the appropriate RS markers, and *in vitro* analysis for RS using enzymatic reactions can be applied in the later generations. Before a high RS cultivar is released, *in vivo* experiments should be carried out for verification. However, limited information is available for RS related genes or QTLs in crops with only one report in rice and wheat. Using the RS111 mutant as the high RS parent, two SSR markers each on chromosome (chr) 8 and 6 were reported to be associated with RS in one F_2_ population, but only the SSR on chr 8 was verified in another F_2_ population in rice [28]. A SSR marker *Xbarc59* conferring high RS content in wheat was identified using bulked segregant analysis (BSA) [29].

Using the Jiangtangdao 1 RS mutant, our objective in the present study was to map, verify and elucidate the mutated gene for resistant starch in rice.

## Materials and Methods

### Plant materials

The mutant Jiangtangdao 1, which is high in RS content, was identified from a doubled haploid (DH) population derived from ‘Huaqingdao’, an early-maturing *japonica* cultivar [20]. Jiangtangdao means reducing-sugar rice in Chinese. The mutagenesis using 0.015% of N- methylnitrosourea (NMU) solution was performed on young panicles of Huaqingdao eight hours after fertilization for 45 min. Following the mutagenesis, anther culture was carried out with standard protocol and chromosome doubling happened naturally during the field-grow out after the plants were regenerated [30].

In 2008, Jiangtangdao 1 was crossed with an *indica* cultivar, ‘Miyang 23’ in the experimental farm of Shanghai Academy of Agricultural Sciences (SAAS), and the resultant F_1_ was grown in Hainan winter nursery. In 2009, 178 F_2_ individuals and both parents were grown at 26×16 cm at the SAAS. From each F_2_ individual, leaf tissue was harvested for genotyping and seeds were harvested for phenotyping of RS. Both genotyping and phenotyping of the F_2_ population were used for primary mapping of putative quantitative trait loci (QTL) for RS. Subsequently, a F_2_ plant heterozygous in the target region identified in the primary mapping was selected to generate four F_3∶4_ family populations for fine mapping.

### Measurement of amylose and resistant starch

Apparent amylose content (AAC) was determined using a previously described colorimetric method with potassium iodide [31]. RS was measured according to the method of Megazyme RS assay kit (Megazyme, Co. Wicklow, Ireland), which has been widely used for RS determination in crops [32]. The grain sample was treated with 10 mg/ml pancreatic α-amylase and 3 U/ml amyloglucosidase (AMG) enzymes for hydrolysis and solubilization of non-resistant starch. After the enzymatic reaction was terminated by adding a 99% ethanol solution, RS was recovered as a pellet by centrifugation (approx. 3.000 rpm, 10 min). RS in the pellet was dissolved in 2 M KOH before the reacted solution was repeatedly washed and decanted. Then, starch in the solution was quantitatively hydrolyzed to glucose with AMG. D-glucose was measured with glucose oxidase/peroxidase (GOPOD) reagent at 510 nm wavelength against the reagent blank. All analyses were repeated three times for error control.

### DNA extraction, SSR primers selection and PCR

Total genomic DNA was extracted from fresh leaves of each offspring plant using the cetyltrimethylammonium bromide (CTAB) method with minor modifications [33]. A total of 541 SSR markers distributed across 12 chromosomes were selected from Gramene (http://www.gramene.org/markers/) to determine polymorphism between the parents Jiangtangdao 1 and Miyang 23. The identified markers were employed for primary mapping using the F_2_ population.

The volume of PCR reaction was 20 µl, containing 2 µl 10×PCR buffer (100 mM Tris-HCl pH 8.0, 15 mM MgCl_2_, 500 mM KCl, 1% TritonX-100), 0.2 mM dNTPs, 0.2 µM forward and reverse primer, 50–100 ng genomic DNA and 0.625 U Taq polymerase. The PCR products were separated on 8% polyacrylamide gel. Bands were detected using a rapid silver staining method [34].

### Marker development

For high-resolution genetic mapping, sequence tagged site (STS) and insertion-deletion (InDel) markers were developed based on DNA sequence difference between 9311(*O. sativa indica*) and Nipponbare (*O. sativa japonica*). Genome sequences for *indica* 9311 and *japonica* Nipponbare were downloaded from BGI (http://rise2.genomics.org.cn/page/rice/index.jsp) and NCBI (http://www.ncbi.nlm.nih.gov/), respectively. The downloaded sequences were aligned using the SeqMan program (A component of DNAStar Lasergene 8.0) for identification of InDel markers. Primers flanking the identified InDels were designed using the online software Primer 3 (http://fokker.wi.mit.edu/primer3/input.htm). In this study, seven polymorphic InDel markers were identified (Table 1).

**Table 1:** Primer sequence of InDel markers developed for fine mapping of *sbe3-rs* flanked by RM13313 and RM8254.

Marker name	Forward primer (5′-3′)	Reverse primer (5′-3′)	Product size (bp) in Nipponbare/9311
InDel 1	CCATCTCCGGTTCGATTGAT	GTCAGCCACGCGACACTC	250/227
InDel 2	CCATGATGCAACGTGTTTTT	GACAATGCCAATGTAGCAGGT	270/258
InDel 3	TCTGACCAAAACCGTCACTG	TGTAACCACACGGCTGAGTC	210/190
InDel 4	CATGCCAATTTCTCGTCTTG	GGCATCTTAATTTGCCGTTA	245/267
InDel 5	AGCAGAGGGAGAAGCTAGGG	GGCATCATCCTGCTTTTGTT	222/240
InDel 6	CCCATGGCATCACACGAG	TGGTTTTCATCTCTATTGGGAAA	244/267
InDel 7	CCGGACAATGCACATATTGA	GGCAAACTTGGGAGATGAAA	203/180

### Analysis of DNA markers and QTL mapping

Genetic linkage maps were constructed using data from the F_2_ and F_4_ populations, respectively. The RS values along with either parental allele pattern of each individual in the populations were applied to Joinmap 4.0 software [35] to calculate marker distances and assign the linked markers to appropriate linkage groups. QTL parameters (location, effect and test statistics) of all putative QTL were estimated using the multiple QTL model mapping (MQM) with MapQTL 6.0 (http://www.kyazma.nl/index.php/mc.MapQTL). The logarithm of odds (LOD) score >3.0 was used to claim significant QTL and interactions for RS in this study.

Fine mapping of major QTL was conducted using 656 individuals from 4 F_3∶4_ families where genetic exchange occurred between Miyang 23 and Jiangtangdao 1 for the identified RS QTL. The developed STS and InDel markers in the identified region were employed for the fine mapping. After the flanking markers were identified during the fine mapping, open reading frames (ORFs) and potential exon/intron boundaries between the flanking markers were screened using the Rice Genome Annotation Project–MSU Release 7 (http://rice.plantbiology.msu.edu/cgi-bin/gbrowse/rice/) based on the defined physical locations. All the ORFs and their functional products in the region were analyzed to predict the candidate gene based on the structures and functions of known starch synthesis genes.

### Isolation of the candidate gene and identification of the mutation site

Total genomic DNA was extracted from leaf tissue of both wild type Huaqingdao and mutant Jiangtangdao 1 using the CTAB method. The annotated coding sequences in the identified candidate gene were targeted for DNA sequencing using the genomic DNA. The primer pairs for the DNA sequencing were designed using the exon coverage according to the sequence of the candidate gene. The PCR amplification mixture contained 2.5 units Ex Taq polymerase (TaKaRa, Dalian, China), 10×Ex Taq buffer, 0.2 mM dNTP, 0.2 µM each of primer and 100 ng of genomic DNA in a final volume of 50 µl. Cycling conditions were 35 cycles of 1 min at 94°C, 1 min at 56°C and 1.5 min at 72°C followed by 10 min at 72°C for final extension. The PCR products were sequenced by Sangon Biotech (http://www.biospace.com/News/sangon-biotech-bio-basic-inc-bbi-closes-10-million/189482). Sequences were assembled using SeqMan program (http://www.dur.ac.uk/stat.web/Bioinformatics/seqman.htm). The sequence of *sbe3-rs* was deposited in the GenBank (http://www.ncbi.nlm.nih.gov/genbank/) with accession number JQ937272 (BankIt1530608 sbe3-rs JQ937272).

After the mutation site was confirmed by comparative analysis of sequencing results between the RS mutant and its wild type, a PCR-based restriction digestion assay was designed to verify the mutation gene. A 571-bp fragment was amplified by PCR using genomic DNA as a template. The primer sequences for PCR-*Spe*I were F: ATGTGATGTGCTGGATTTGG and R: TGTGGTTTTCATACCGTTCTTA. The PCR products were digested by a CAPS (Cleaved Amplified Polymorphic Sequence) marker for *Spe*I (TaKaRa, Dalian, China). The reaction mixtures contained 17.5 µl of PCR product, 2 µl 10× M buffer, 0.5 µl *Spe*I in a total volume of 20 µl and incubated at 37°C for 5 h. Digested products were separated on 1% agarose gel for genotyping of individuals. Finally, the designed marker for the digestion was employed to run the entire F_2_ population for a confirmation of the identified gene using co-segregation of digestion pattern and RS content.

## Results

### Variation of resistant starch (RS) content

**Figure 1:**
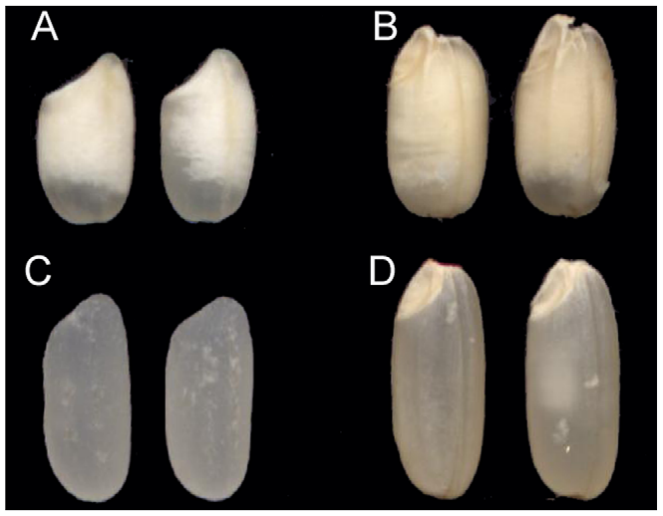
Comparison of grain chalkiness and transparency between Jiangtangdao 1 and Miyang 23. **A, Milled rice of Jiangtangdao 1.** B, Brown rice of Jiangtangdao 1. C, Milled rice of Miyang 23. D, Brown rice of Miyang 23.

**Figure 2:**
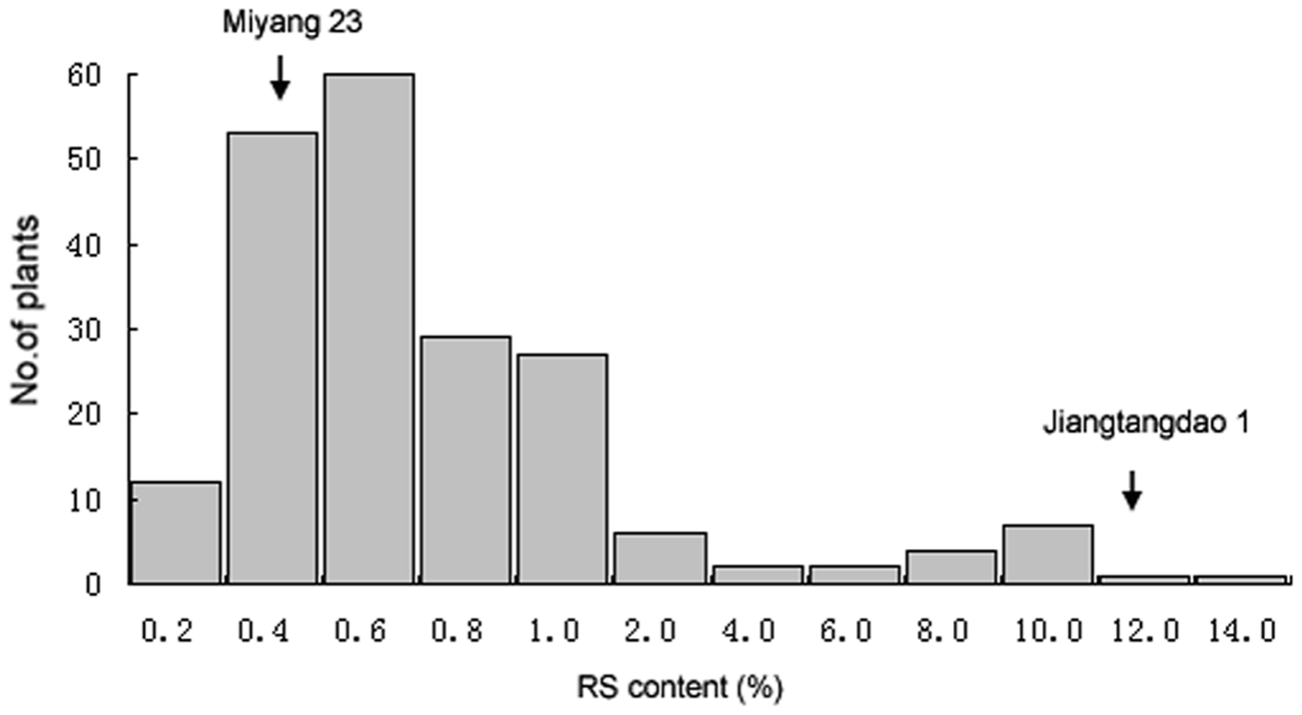
Variation of resistant starch content in the F_2_ population derived from Jiangangdao 1 and Miyang 23.

Parent Jiangtangdao 1 is high in both contents of RS (11.67±0.43%) and apparent amylose content (AAC) (31.10±0.15%). Its RS was 28 times that of the other parent Miyang 23 (0.41±0.16%), and its AAC was two times that of Miyang 23 (15.13±0.05%). Jiangtangdao 1 had an almost-completely opaque endosperm due to increased chalkiness, while the grain of Miyang 23 was quite transparent (Figure 1A, B). In the F_3_ families, the majority of individuals were in the low RS region of 0.4–1.0%, which shaped a long and flat tail extending from 4.0 to 13.67% of RS (Figure 2). This distribution indicated that the responsible QTL were few for RS inheritance, and the mutated high RS locus was recessive to regular-low RS. Few QTL traits usually are less affected by the environment than many QTL traits, evidenced by small standard deviation (0.43%) of RS in Jiangtangdao 1 [36].

### Primary mapping

**Figure 3:**
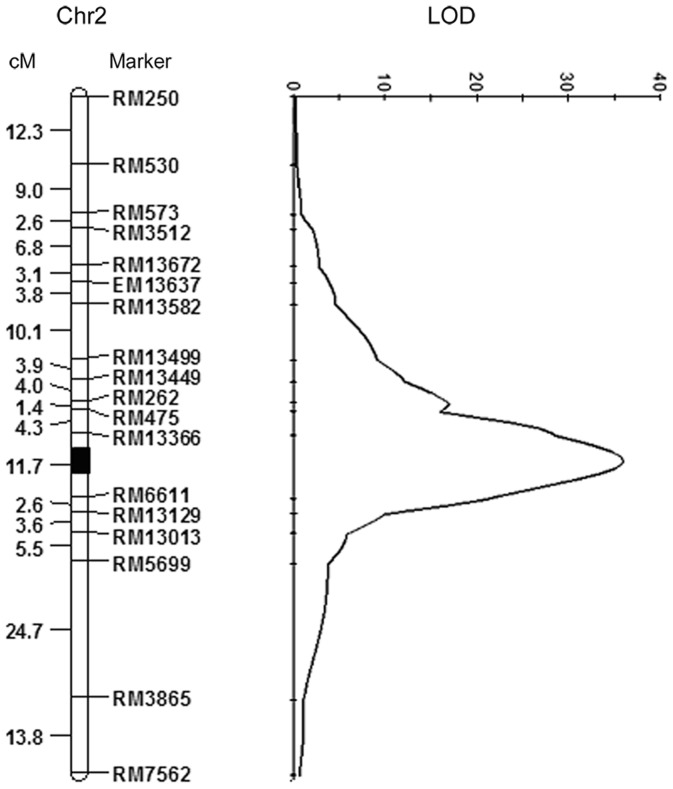
Genetic linkage map of rice resistant starch on chromosome 2 for *sbe3-rs*, genetic distance (Kosambi, centiMorgan) and SSR marker name on the left and right of the chromosome, respectively and highlighted region for the estimated position of *seb3-rs*.

In total, 106 out of 541 SSR markers were polymorphic between Jiangtangdao 1 and Miyang 23. These markers covered 1204.1 cM on the rice genome. Average marker coverage was approximately one marker every 11.4 cM. Based on RS data from 178 F_2_ individuals with the 106 SSRs, only one QTL on chromosome (chr) 2 was identified (Figure 3). This QTL saturated by 18 markers, appeared to be a major QTL as indicated by a high LOD value of 36. The major QTL explained 60.4% of total variation in the F_2_ population of Jiangtangdao 1 and Miyang 23 for RS with additive effect of −3.2611 and dominant effect of −3.1314. Thus, this locus was designated *sbe3-rs* according to QTL nomenclature [37]. The *sbe3-rs* locus was flanked by RM6611 and RM13366 with a genetic distance of 11.7 cM and a physical distance of 2.2 Mb, approximately.

### Fine mapping of *sbe3-rs*

**Figure 4:**
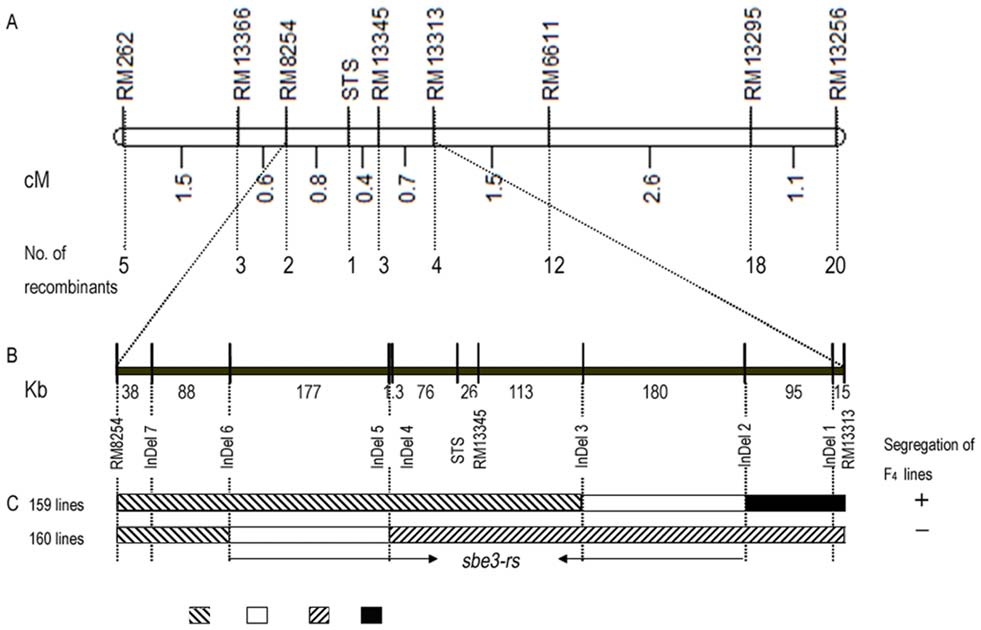
Schematic representation of the region on rice chromosome 2 harboring *sbe3-rs*. A: High-resolution genetic map built with eight SSRs and a STS markers: numbers between the markers indicate genetic distance (cM), and the number of recombinants was among extreme low RS content plants. B: Physical maps between SSR marker RM8254 and RM 13313. C: Recombinant genotypes and their corresponding phenotypes. +, indicates RS content segregating in F_4_ line; − indicates no segregation in F_4_ line. <$>\raster(85%)="rg2"<$>: Genotypes between two flanking markers are heterozygotes; □: Break point of the recombination; <$>\raster(85%)="rg1"<$>: Miyang 23 genotype and ▪: Jiangtangdao 1 genotype.

Four F_3_ plants derived from a recombinant F_2_ plant were identified as heterozygous in the region flanked by RM6611 and RM13366 (Table 2). Selfed seeds of these recombinant F_3_ plants generated 656 F_4_ individuals (210 plants of F_3_ family 156,86 plants of family 157,208 plants of family 159, and 152 plants of family 160) for fine mapping. Screening all the available SSR markers in Gramene (http://www.gramene.org/) within the flanking region of RM6611 and RM13366 identified five SSRs polymorphic between the parents, RM13256, RM13295, RM13313, RM13345 and RM8254, plus a STS marker. Fine mapping with these markers and RS data of 656 F_4_ individuals narrowed the region harboring *sbe3-rs* to a physical distance of 809.4 kb between RM13313 and RM8254 (Figure 4A).

**Table 2:** Genotypes of four recombinant F_3_ individuals for putative resistant starch ***sbe3-rs*** flanked by RM6611 and RM13366: A – ‘Miyang 23’, B – ‘Jiangtangdao 1’ and H – heterozygous.

F_3_ No	RM5699	RM6611	RM13366	RM475	RM262	RM3512
156	A	H	H	H	H	A
157	A	H	H	H	H	A
159	A	B	H	H	H	A
160	A	A	H	H	H	A

To further validate the mapping result, seven polymorphic InDel markers, InDel 1–7, were developed for fine mapping *sbe3-rs* (Table 2). We analyzed RS variation and the seven InDel markers among 360 F_4_ individuals derived from two F_3_ families, family 159 and 160. RS segregated in F_3∶4_ family 159, which genotypes for markers between RM13313 and InDel2 were same to the high RS parent Jiangtangdao 1 (Figure 4B). Thus, *sbe3-rs* was determined not to be between RM13313 and InDel2. On the other hand, RS did not segregate in F_3∶4_ family 160, which genotypes for markers between RM13313 and InDel5 matched the low RS parent Miyang 23. Thus, the region between Indel6 and RM13366 was rejected as the location for *sbe3-rs*. Both rejections on each end suggested that *sbe3-rs* was between InDel2 and InDel6, which physical distance was narrowed to 573 kb.

### Verification and mutation identification using sequencing the candidate gene

**Figure 5:**
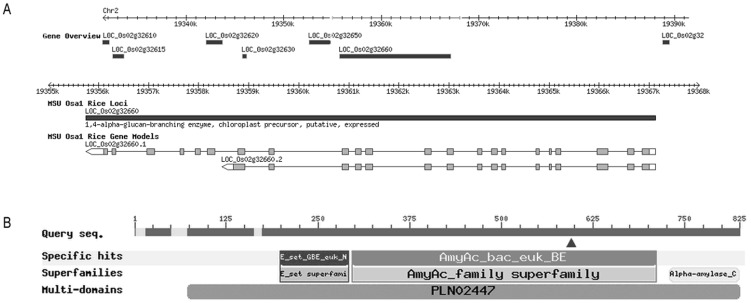
Physical location and domain structure of SBE3 gene. A: Diagram of genomic interval within the target region on chromosome 2 of Nipponbare. B: Conserved domain of predicted candidate gene *SBE3* (LOC_Os02g32660). E_Set superfamily: early set domain associated with the catalytic domain of sugar utilizing enzymes; AmyAc_Family superfamily: alpha amylase catalytic domain; Alpha-amylase_C: alpha amylase, C-terminal all-beta domain; and ▴: Mutation site.

**Figure 6:**
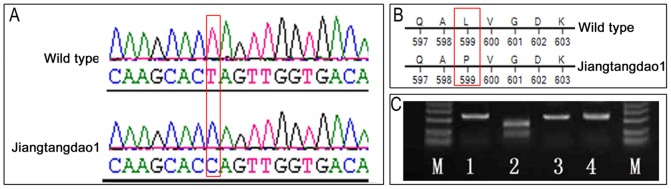
Sequence confirmation of the PCR products. A: Nucleotide ‘T’ of the wild type was substituted by ‘C’ of the mutant Jiangtangdao 1; B: The substitution resulted in a missense from Leu-599 of the wild in *SBE3* coding region for 599 amino acid to Pro-599 of Jiangtangdao 1 in *sbe3-rs* coding for high resistant starch; and C Because the point mutation resulted in the loss of a restriction enzyme site *Spe*I, *sbe3-rs* of the mutant was not digested with *Spe*I, while *SBE3* of the wild type was (M: marker DL2000; Lane 1–2: wild type before and after digestion; and lane 3–4: Jiangtangdao 1 before and after digestion).

From Rice Genome Annotation Project (http://rice.plantbiology.msu.edu/index.shtml), 86 genes (Table S1) were predicted in the region where *sbe3-rs* was flanked by InDel2 and InDel6. Among the 86 genes, starch branching enzyme (*SBE3*, LOC_Os02g32660) was the only candidate gene known to be associated with starch synthesis and could be a top candidate for the resistant starch locus in rice (Figure 5A). The *SBE3* candidate gene, which has 3 protein domains, encodes a branching enzyme to introduce α-1,6 bonds that is essential for the formation of amylopectin (Figure 5B). The genomic DNA sequence of *SBE3* includes 11,380 nucleotides with 22 exons. The *SBE3* gene consisted of 2478 bp encoding a predicted protein with 825 amino acid residues. We designed nine pairs of primers to sequence genomic DNA of both mutant Jiangtangdao 1 and its wild type Huaqingdao for the exons of *SBE3* gene (Table 3). Comparison of the sequencing results displayed that a single nucleotide substitution (T to C) occurred within the coding region of *SBE3* in the wild type (Figure 6A). This nucleotide substitution caused a missense base change from a CTA (Leu) to CCA (Pro) at codon 599 in exon 16 (Figure 6B). Analysis of the predicted products from gene *SBE3* demonstrated that the mutated amino acid was on protein domain clo7893, an Alpha amylase catalytic domain family.

**Table 3:** Primers used for sequencing the putative gene *SBE3* (LOC_Os02g32660).

Name	Sequence (5′-3′)	Exon coverage	Product size (bp)
SBE3-P1F	GTGAGGAGGGTTTAGGTGGAAG	Exon 1–3	1201
SBE3-P1R	TTGTGAAAGACTGAACAGATGGA		
SBE3-P2F	TAATGGGTTTTAGACCTTGCTGA	Exon 4–6	1209
SBE3-P2R	CCTATCAAGTCCACAAAAACTGC		
SBE3-P3F	TCGGTTTTGATGCTACTGTAGTG	Exon 7–9	1174
SBE3-P3R	CAAATGGTAGGCATGGTGTTATT		
SBE3-P4F	TTTGCACATTCGTCAACAATTAG	Exon 10–11	1345
SBE3-P4R	GGAACCAAACATTAATCCACAAA		
SBE3-P5F	GTGACATCTTTTGTTGGCTTTCT	Exon 12–14	1070
SBE3-P5R	AATTTCTTGAACCAGCGACATAA		
SBE3-P6F	ACAGGGAGAAAGGAAGAAAAATG	Exon 15	789
SBE3-P6R	CATGGTCAAGGAAACCAATGTAT		
SBE3-P7F	AGTTTGACTTGGCAACGTTACAT	Exon 16–18	1301
SBE3-P7R	GAATTGTCATAATGGGACCTTCA		
SBE3-P8F	AACTTTCTTTGCTCTGTCACCTG	Exon 19–20	1192
SBE3-P8R	AGGATCCAGACCTAGGACTATCG		
SBE3-P9F	TACCTACACTAGTCGTGCCCATT	Exon 21–22	551
SBE3-P9R	TGTCTAGTCTATCCGGCATTCAT		

**Figure 7:**
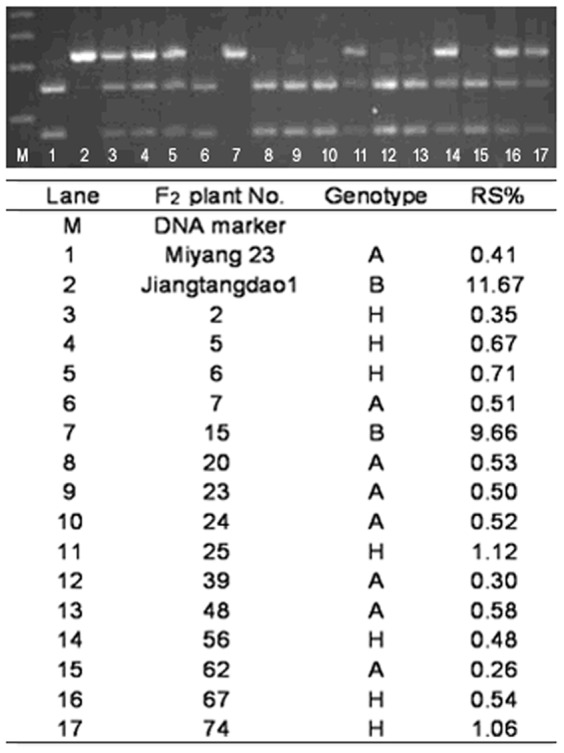
Verification of the CAPS marker using a part of F_2_ individuals.

The sequence result was also verified by restriction enzyme *Spe*I digestion (TakaRa, Dalian, China). The mutant Jiangtangdao 1 was not digested by the CAPS marker for *Spe*I (Figure 6C, lane 4), while the wild type was (Figure 6C, lane 2). Among 178 F_2_ plants, 70 had Miyang 23 genotype which RS contents ranged from 0.25 to 0.73%, 17 had Jiangtangdao 1 genotype which RS contents ranged from 4.56 to 12.73%, and the remaining 91 had heterozygote genotype which RS contents ranged from 0.35 to 2.19% (Figure 7). Obviously, plant genotype revealed by the CAPS marker co-segregated with RS phenotype, and the co-segregation further supported *sbe3-rs* responsible for RS in rice.

## Discussion

### Cultivar improvement for resistant starch

Mapping genes or QTL and development of markers are essential for marker-assisted breeding (MAB), especially for RS for which assessment is extremely difficult *in vivo* [24], and questionable *in vitro* [24], [25]. We 1) mapped *sbe3-rs*, a recessive gene for high RS in Jiangtangdao 1 mutated from *SBE3* using a F_2_ population, 2) finely mapped *sbe3-rs* using F_3∶4_ populations, 3) conducted a verification by comparatively sequencing the putative region for *sbe3-rs* and *SBE3* in the mutant and wild type, respectively, and 4) further verified the mapped gene using the products from *sbe3-rs* and *SBE3* restricted by a CAPS marker. The CAPS marker was developed based on the nucleotide difference in coding region between *sbe3-rs* and *SBE3*. Because the point mutation resulted in the loss of a restriction enzyme site *Spe*I, *sbe3-rs* was not digested by the CAPS marker for *Spe*I while *SBE3* was. Co-segregation of the *sbe3-rs* genotype with high RS phenotype demonstrated that the CAPS marker could be used for MAB to screen for elevated RS in rice reliably and accurately.

Because of its effective control of glycemic index (GI) for diabetic patients, Jiangtangdao 1 with high RS content has been commercially developed. However, its commercialization has been largely restricted by its productivity since Jiangtangdao 1 is prone to lodging and diseases in addition to low milling yield. The low productivity along with high demands in the market has resulted in a high price of Jiangtangdao 1. Employing marker-assisted breeding techniques with the developed CAPS marker should improve the productivity of Jiangtangdao 1 by improving the efficiency of breeding with a reliable and accurate selection, and increase its availability to serve more diabetic patients subsequently.

### *SBE3* and *sbe3-rs* in starch synthesis

Starch branching enzymes, such as the one encoded by *SBE3*, introduce a-1,6 linkages into starch that are critical for the formation of amylopectin [11]. There are two families of starch branching enzymes in rice based on primary structure and functional analysis. SBE3 belongs to family A, which is responsible for a unique aspect of amylopectin biosynthesis and structure in rice development [38]. Lack of SBE I combined with a lack of SBE IIb has been reported to produce a much more branched starch without any change in the property of amylose [39]. It has been reported that rice deficient in SBE3 produces apparent amylose up to 29%–35% [40].

Genetic modification of *SBE* has demonstrated to successfully increase resistant starch and amylose in cereals. Using genetic transformation technology, wheat produces up to 80% amylose along with substantially high resistant starch when *SBE IIa* is knocked out [10]. Similarly, maize produces up to 80% amylose with high resistant starch when *SBE IIb* is knocked out [41]. The differences between wheat and maize are due to the fact that SBE IIa is the predominant isoform in wheat grain, while SBE IIb is the predominant isoform in maize [10]. A high-amylose transgenic rice line (TRS) modified by antisense RNA inhibition of *SBE1* and *SBE3* yields substantially high resistant starch [21], which effectively improves large bowel health in rats [42].

By comparing the nucleotide sequences of *SBE3* and *sbe3-rs*, we located the recessive point mutation in exon 16 where the nucleotide T in the wild type was substituted by C in mutant Jiangtangdao 1. This substitution resulted in a missense in *SBE3* coding region for 599 amino acid from Leu-599 of the wild to Pro-599 of Jiangtangdao 1. The mutation occurred in the protein domain clo7893 as Alpha amylase catalytic domain family. It has been reported that the proline residues may cause a bend in the helix [43], evidenced by their presence on the solvent exposed face of each helix [44]. As a result, these residues play an important role in determination of local conformation for protein three-dimensional structure [45]. Therefore, the change of missense base, in our case from Leu-599 coded by *SBE3* to Pro-599 coded by, *sbe3-rs* may impact the protein conformation of SBE3. However, a functional complementation of the *sbe3-rs* gene in Jiangtangdao 1 with the wild type copy of the *SBE3* gene using transgenic technology should be conducted for an absolute conclusion on resistant starch in rice.

## Supporting Information

Table S1 **The 86 genes and their putative functions in the region between InDel2 and InDel6 where** ***sbe3-rs*** **was located for rice resistant starch, predicted in Rice Genome Annotation Project (** http://rice.plantbiology.msu.edu/index.shtml **).**
(DOC)
