# Supplementary material for: A Putative Gene sbe3-rs for Resistant Starch Mutated from SBE3 for Starch Branching Enzyme in Rice (Oryza sativa L.)
Source: PLoS One. 2012 Aug 24;7(8):e43026. doi: 10.1371/journal.pone.0043026 (PMC3427327; doi:10.1371/journal.pone.0043026)
Supplement: Table S1 — The 86 genes and their putative functions in the region between InDel2 and InDel6 where sbe3-rs was located for rice resistant starch, predicted in Rice Genome Annotation Project ( http://rice.plantbiology.msu.edu/index.shtml ). (DOC) [file pone.0043026.s001.doc]

***Supplemental file as below***

**Supplemental Table S1** The 86 genes and their putative functions in the region between InDel2 amd InDel6 where *sbe3-rs* was located for rice resistant starch, predicted in Rice Genome Annotation Project (<http://rice.plantbiology.msu.edu/index.shtml>).

| Locus Identifier | Putative Function |
| --- | --- |
|
| LOC_Os02g32090 | retrotransposon protein, putative, unclassified, expressed |
| LOC_Os02g32100 | retrotransposon protein, putative, unclassified, expressed |
| LOC_Os02g32110 | exostosin family domain containing protein, expressed |
| LOC_Os02g32120 | cytochrome b-c1 complex subunit Rieske, mitochondrial precursor, putative, expressed |
| LOC_Os02g32130 | expressed protein |
| LOC_Os02g32140 | AP2 domain containing protein, expressed |
| LOC_Os02g32160 | copine, putative, expressed |
| LOC_Os02g32170 | transposon protein, putative, unclassified, expressed |
| LOC_Os02g32180 | expressed protein |
| LOC_Os02g32190 | expressed protein |
| LOC_Os02g32200 | thioesterase family protein, putative, expressed |
| LOC_Os02g32210 | expressed protein |
| LOC_Os02g32220 | retrotransposon protein, putative, unclassified, expressed |
| LOC_Os02g32230 | retrotransposon protein, putative, unclassified, expressed |
| LOC_Os02g32240 | retrotransposon protein, putative, unclassified, expressed |
| LOC_Os02g32250 | retrotransposon protein, putative, unclassified, expressed |
| LOC_Os02g32270 | expressed protein |
| LOC_Os02g32280 | expressed protein |
| LOC_Os02g32290 | expressed protein |
| LOC_Os02g32300 | retrotransposon protein, putative, unclassified, expressed |
| LOC_Os02g32310 | expressed protein |
| LOC_Os02g32320 | expressed protein |
| LOC_Os02g32340 | Spc97 / Spc98 family protein, putative, expressed |
| LOC_Os02g32350 | TUDOR protein with multiple SNc domains, putative, expressed |
| LOC_Os02g32360 | transposon protein, putative, CACTA, En/Spm sub-class, expressed |
| LOC_Os02g32370 | inositol hexaphosphate kinase, putative, expressed |
| LOC_Os02g32380 | expressed protein |
| LOC_Os02g32390 | expressed protein |
| LOC_Os02g32400 | 50S ribosomal protein L19, chloroplast precursor, putative, expressed |
| LOC_Os02g32410 | expressed protein |
| LOC_Os02g32420 | protein binding protein, putative, expressed |
| LOC_Os02g32430 | WD domain, G-beta repeat domain containing protein, expressed |
| LOC_Os02g32440 | expressed protein |
| LOC_Os02g32450 | expressed protein |
| LOC_Os02g32460 | ThiF family domain containing protein, putative, expressed |
| LOC_Os02g32465 | expressed protein |
| LOC_Os02g32469 | expressed protein |
| LOC_Os02g32480 | expressed protein |
| LOC_Os02g32490 | AMP-binding enzyme, putative, expressed |
| LOC_Os02g32504 | heparan-alpha-glucosaminide N-acetyltransferase, putative, expressed |
| LOC_Os02g32520 | ERD1 protein, chloroplast precursor, putative, expressed |
| LOC_Os02g32530 | SAM domain family protein, expressed |
| LOC_Os02g32540 | ubiquitin carboxyl-terminal hydrolase, family 1, putative, expressed |
| LOC_Os02g32550 | expressed protein |
| LOC_Os02g32560 | retrotransposon protein, putative, LINE subclass, expressed |
| LOC_Os02g32570 | SNF2 family N-terminal domain containing protein, expressed |
| LOC_Os02g32580 | expressed protein |
| LOC_Os02g32590 | HSF-type DNA-binding domain containing protein, expressed |
| LOC_Os02g32600 | expressed protein |
| LOC_Os02g32610 | protein kinase domain containing protein, expressed |
| LOC_Os02g32615 | expressed protein |
| LOC_Os02g32620 | PAN domain-containing protein At5g03700 precursor, putative, expressed |
| LOC_Os02g32630 | expressed protein |
| LOC_Os02g32650 | expressed protein |
| LOC_Os02g32660 | 1,4-alpha-glucan-branching enzyme, chloroplast precursor, putative, expressed |
| LOC_Os02g32680 | lectin receptor-type protein kinase, putative, expressed |
| LOC_Os02g32690 | pleiotropic drug resistance protein 15, putative, expressed |
| LOC_Os02g32700 | autophagy-related protein, putative, expressed |
| LOC_Os02g32710 | expressed protein |
| LOC_Os02g32720 | hypothetical protein |
| LOC_Os02g32730 | neutral/alkaline invertase, putative, expressed |
| LOC_Os02g32740 | SNARE domain containing protein, putative, expressed |
| LOC_Os02g32750 | glycosyltransferase, putative, expressed |
| LOC_Os02g32760 | 60S acidic ribosomal protein, putative, expressed |
| LOC_Os02g32770 | cytochrome P450, putative, expressed |
| LOC_Os02g32780 | pentatricopeptide, putative, expressed |
| LOC_Os02g32790 | hypothetical protein |
| LOC_Os02g32814 | heavy metal-associated domain containing protein, expressed |
| LOC_Os02g32840 | zinc finger A20 and AN1 domain-containing stress-associated protein, putative, expressed |
| LOC_Os02g32850 | expressed protein |
| LOC_Os02g32860 | poly synthetase 3, putative, expressed |
| LOC_Os02g32870 | retrotransposon protein, putative, unclassified, expressed |
| LOC_Os02g32880 | retrotransposon protein, putative, Ty3-gypsy subclass, expressed |
| LOC_Os02g32890 | transposon protein, putative, CACTA, En/Spm sub-class, expressed |
| LOC_Os02g32900 | expressed protein |
| LOC_Os02g32910 | retrotransposon protein, putative, unclassified, expressed |
| LOC_Os02g32920 | expressed protein |
| LOC_Os02g32930 | bile acid sodium symporter, putative, expressed |
| LOC_Os02g32940 | expressed protein |
| LOC_Os02g32950 | RCN2 Centroradialis-like1 homologous to TFL1 gene; contains Pfam profile PF01161: Phosphatidylethanolamine-binding protein, expressed |
| LOC_Os02g32960 | expressed protein |
| LOC_Os02g32970 | hydrolase, alpha/beta fold family protein, putative, expressed |
| LOC_Os02g32980 | Cupin domain containing protein, expressed |
| LOC_Os02g32990 | expressed protein |
| LOC_Os02g33000 | glycosyl hydrolases family 17, putative, expressed |
